# Effect of Rolling Route on Microstructure and Tensile Properties of Twin-Roll Casting AZ31 Mg Alloy Sheets

**DOI:** 10.3390/ma9060433

**Published:** 2016-06-01

**Authors:** Dan Luo, Yue Pan, Hui-Yuan Wang, Li-Guo Zhao, Guo-Jun Liu, Yan Liu, Qi-Chuan Jiang

**Affiliations:** 1 Key Laboratory of Automobile Materials of Ministry of Education & School of Materials Science and Engineering, Nanling Campus, Jilin University, No. 5988 Renmin Street, Changchun 130025, China; danluo198709@gmail.com (D.L.); panyue0503@163.com (Y.P.); chenl11@jlu.edu.cn (L.-G.Z.); liuguojun@jlu.edu.cn (G.-J.L.); jqc@jlu.edu.cn (Q.-C.J.); 2 Key Laboratory of Bionic Engineering (Ministry of Education), Jilin University, Changchun 130025, China

**Keywords:** magnesium alloy, texture, rolling route, mechanical properties

## Abstract

Twin-roll casting AZ31 Mg alloy sheets have been fabricated by normal unidirectional-rolling, head-to-tail rolling, and clock-rolling, respectively. It has been demonstrated that head-to-tail rolling is the most effective to refine the microstructure and weaken the basal texture among the three rolling routes. Excellent integrated tensile properties can be obtained by the head-to-tail rolling. The yield strength, ultimate tensile strength, and plastic elongation are 196 MPa, 301 MPa, and 28.9%, respectively. The strength can benefit from the fine grains (average value of 4.0 μm) of the AZ31 alloy processed by the head-to-tail rolling route, while the excellent plastic elongation is achieved owing to the weakened basal texture besides the fine grains. Results obtained here can be used as a basis for further study of some simple rolling methods, which is critical to the development of Mg alloys with high strength and plasticity.

## 1. Introduction

Twin-roll casting is an effective method to produce metal alloys while significantly reducing costs [1,2,3]. However, centerline segregation and coarse columnar dendritic grains form during the twin-roll casting process, which has a deleterious effect on the strength and ductility due to the limited quality in Mg alloys [3]. Sequential warm rolling has been developed to refine grains of Mg alloys after the twin-roll casting process [4,5]. However, such a method often results in a strong basal texture [4]. The basal texture with most grains in hard orientation is difficult to deform since the resolved shear stress in the basal plane is essentially zero, which leads to stress localization and premature failure [6,7]. Therefore, it is of significant interest to find methods to avoid the development of the basal texture during the rolling deformation process [6].

There are many reports on the weakening of the basal texture intensity and the inclining of the basal pole obtained by different methods [8,9,10]. Changing the rolling route has been considered to be one of the effective methods to decrease the basal texture strength and enhance the rollability of Mg alloys [11]. Higher strength and elongation can be achieved due to finer grains and weaker basal texture obtained by changing the rolling routes [12]. However, twin-roll casting is still difficult at present, and the related research about different rolling routes of cast-rolling AZ31 Mg alloy sheets have not been investigated thoroughly [6]. In this work, twin-roll casting AZ31 Mg alloy sheets have been manufactured by normal unidirectional-rolling, head-to-tail rolling, and clock-rolling, respectively. The work focuses primarily on the microstructure and tensile properties of the hot-rolled AZ31 alloy sheets, and particular attention was paid to investigate the evolution of texture.

## 2. Experimental Details

The as-received AZ31 Mg alloy sheets with a thickness of 6 mm were fabricated by the twin-roll casting method. The twin-roll casting sheets were cut into rectangular slabs of 40 mm (rolling direction, RD) × 40 mm (transverse direction, TD) × 6 mm (normal direction, ND) and then were homogenized at 430 °C for 3 h before rolling. Afterwards, the slabs were hot-rolled from 6 to 1 mm after eight passes with reduction ratios of ~28%, ~24%, ~22%, ~19%, ~27%, ~9%, ~14% and ~15% successively. The slabs were preheated at 200 °C for 15 min before the first pass and for 10 min before subsequent passes. Finally, the as-rolled samples were annealed at 200 °C for 30 min.

Microstructures were observed by an optical microscope (OM) (Carl Zeiss-Axio Imager A2m, Oberkochen, Germany). The electron backscatter diffraction (EBSD) measurements were performed on a scanning electron microscope (SEM) (Zeiss Supra55 (VP), Oberkochen, Germany) with the software Channel 5. The EBSD was performed at 15 kV, with a tilt angle of 70° and a scan step of 0.6 or 4 μm. The X-ray diffraction (XRD) (D/Max 2500PC, Rigaku, Japan) was employed to analyze the phases using Cu K_α_ radiation in step mode from 20° to 80° with a scanning speed of 4° min^−1^ and an acquisition step of 0.02° (2θ). Samples for OM microstructure observation were firstly ground with 2000 mesh SiC papers, followed by buffing with 0.5 μm diamond pastes, and then chemically etched in acetic picral solution (5 mL acetic acid, 5 g picric acid, 10 mL distilled water, and 80 mL ethanol) for 15 s. Samples for the EBSD microstructure observation were firstly mechanically polished and then followed with argon ion polishing (Gatan Ilion II 697, Pleasanton, CA, USA) at a voltage of 8.0 kV for 15 min, 4 kV for 15 min, and 1 kV for 30 min successively. Tensile samples machined from the as-annealed sheets (a gage size of 30 mm × 10 mm × 0.7 mm) were tested along the RD on a material testing machine (INSTRON 5869, Cambridge, MA, USA) at a strain rate of 1.0 × 10^−3^ s^−1^. Stress–strain curves with good repeatability have been used.

## 3. Results and Discussion

**Figure 1:**
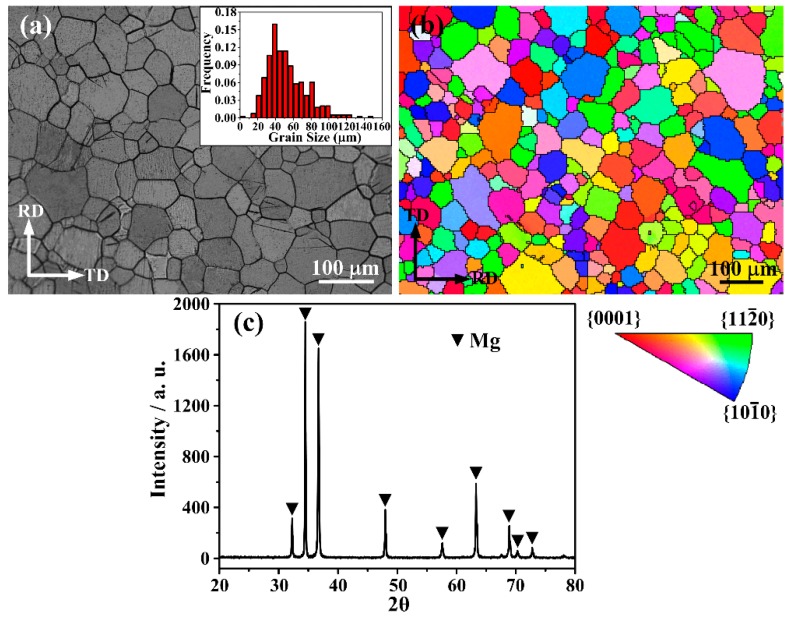
(**a**) Optical micrograph with the top-right corner inset showing a grain size distribution; (**b**) inverse pole figure (IPF) map and (**c**) X-ray diffraction (XRD) pattern of the homogenized AZ31 Mg alloy at 430 °C for 3 h.

The optical microstructure of the homogenized twin-roll casting AZ31 alloy is shown in Figure 1a. It can be seen the grains are coarse and the grain size primarily ranges between 20 and 80 μm (the inset in Figure 1a), and the average grain size is 53 μm. To examine the orientation of the grains, EBSD measurements were performed. It can be seen that the orientation of the grains is random in the inverse pole figure (IPF) map, which indicates the basal texture is weak in the homogenized AZ31 alloy (Figure 1b). The XRD pattern further confirms that the basal texture is very weak in the homogenized AZ31 alloy (Figure 1c). Only α-Mg phase is detected by XRD (Figure 1c), which indicates that eutectic Mg_17_Al_12_ phases are thoroughly solid-dissolved after the homogenization treatment.

**Figure 2:**
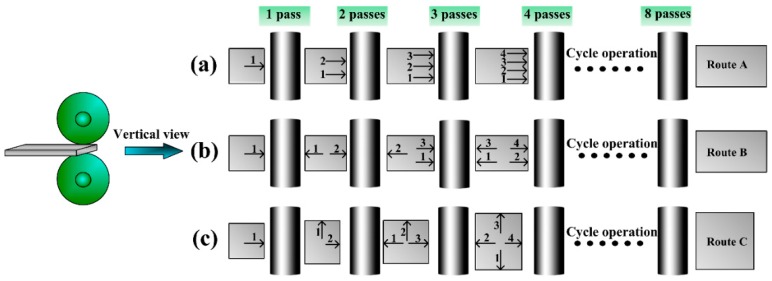
Schematic diagrams of the three rolling methods: (**a**) Route A; (**b**) Route B; and (**c**) Route C.

Figure 2 shows the schematic diagram of the three rolling routes. Route A is unidirectional-rolling, where the rolling direction is always constant (Figure 2a). Route B (head-to-tail rolling), differing from the normal unidirectional rolling, has two rolling directions (Figure 2b). The rolling direction of Route B is changed by 180° repeatedly (Figure 2b). The last one is Route C (clock-rolling), where the rolling direction is changed anticlockwise by 90° after each rolling (Figure 2c).

**Figure 3:**
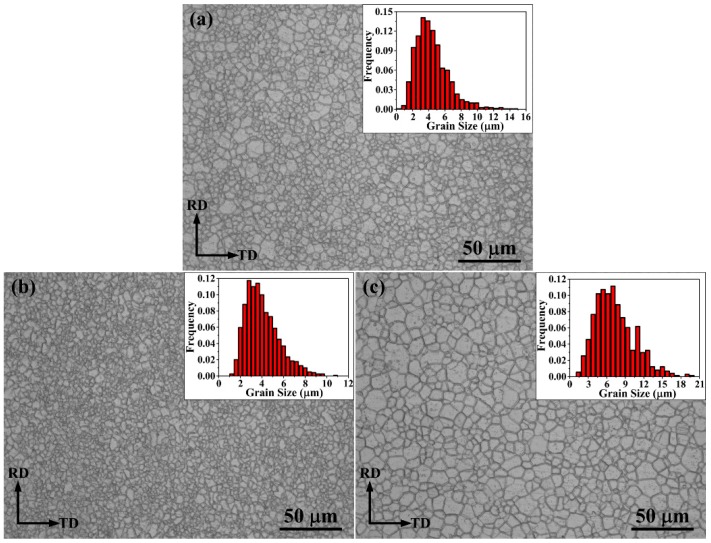
Optical micrographs with the top-right corner insets showing the grain size distribution of the as-annealed AZ31 Mg alloy processed by (**a**) Route A; (**b**) Route B; and (**c**) Route C, respectively.

Optical microstructures of the as-annealed AZ31 alloy processed by Route A, B, and C are presented, respectively in Figure 3a–c. It can be observed that the microstructure of as-annealed samples is completely recrystallized. Fine and equiaxed grains form by the three rolling methods. The average grain sizes are 4.4, 4.0, and 7.3 μm, respectively (Figure 3a–c). Compared with the microstructure of the AZ31 alloy processed by Route A, the one processed by Route B is refined, which is similar to the result of the AZ31 Mg alloy sheets rolled by changing the rolling route (cross-rolling, rotating the specimen by 90° after each rolling step back and forth) in previous research [12]. It has been reported that the grains of the AZ31 alloy processed by cross-rolling are finer than those processed by the unidirectional-rolling (Route A). The strain path can define the microstructure of a sample during the rolling deformation process, and grains usually tend to be elongated towards the rolling direction after each rolling [6]. Dynamic recovery (DRV) can be promoted by the constant change of the microstructure, which in turn influences the behavior of the recrystallization [6]. However, the microstructure processed by Route C consists of more coarse grains compared with the ones processed by Route A and B (Figure 3c), which causes an adverse effect on the grain size of the AZ31 alloy, probably due to relatively weak shear deformation between each rolling pass.

**Figure 4:**
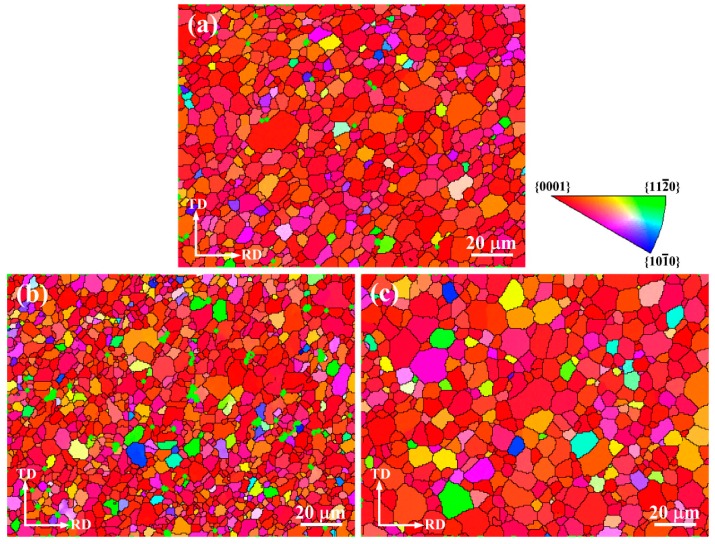
IPF maps of the as-annealed AZ31 Mg alloy processed by (**a**) Route A; (**b**) Route B; and (**c**) Route C, respectively.

To examine the microstructure in detail, IPF maps of the as-annealed AZ31 Mg alloy sheets processed by Route A, B, and C are shown in Figure 4. Color difference of the homogenized AZ31 alloy shows that the orientation of the c-axis of the grains is random (Figure 1b), while the three rolling methods give rise to the rotation of c-axis, and the c-axis of most of the grains are parallel to the normal direction due to the increasingly accumulated strain (Figure 4). Note that grains are refined in the microstructure processed by Route B (Figure 4b), which is consistent with the result from the optical microstructure (Figure 3b).

**Figure 5:**
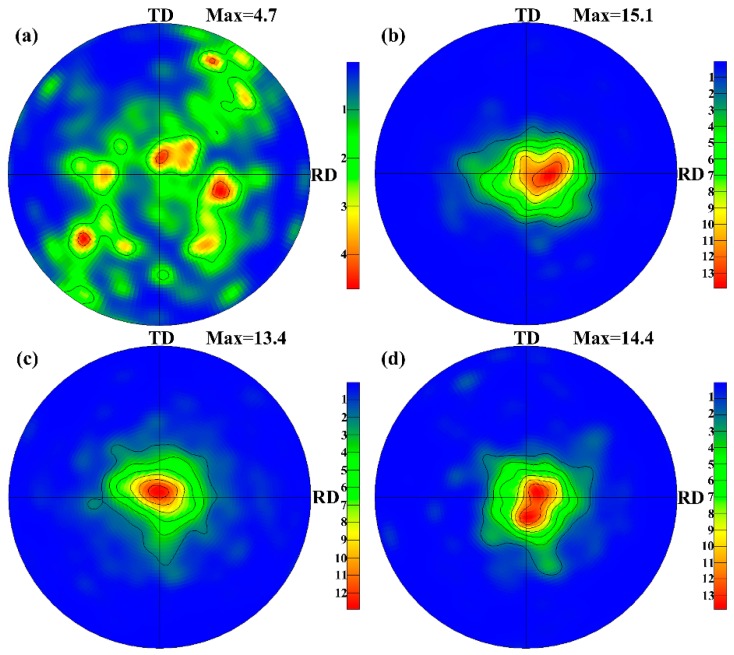
(0 0 0 2) pole figures of the AZ31 Mg alloy before and after the rolling by different routes: (**a**) homogenized; (**b**) Route A; (**c**) Route B; and (**d**) Route C, respectively.

Figure 5 shows the (0002) pole figures of the AZ31 alloy before and after the rolling by different routes. In the initial homogenized AZ31 alloy, the basal texture intensity is 4.7 and it can be seen that disperse texture components form in Figure 5a, which shows that the texture is weak and is consistent with the result from the IPF microstructure in Figure 1b. The basal texture intensity is 15.1, 13.4, and 14.4 for the AZ31 alloy sheets processed by Route A, B, and C, respectively. The AZ31 alloy sheet processed by Route A shows a typical strong basal texture (Figure 5b), where the distribution of the orientation around the normal direction is wider in the rolling direction compared with the one in the transverse direction [12]. The formation of strong basal texture in rolled AZ31 can be attributed to both of the basal <**a**> slip and tensile twinning [13,14,15]. The AZ31 alloy sheet processed by Route B also exhibits typical basal texture, but the texture has been weakened by the constant change of rolling direction in Figure 5c. The AZ31 alloy sheet processed by Route C shows stronger texture than the one processed by Route B, but the texture is still weaker than the one processed by Route A. Therefore, the basal texture can be weakened by both the head-to-tail rolling and clock-rolling. Moreover, note that basal pole tends to split in the Figure 5d. It has been reported that pyramidal <**c** + **a**> slip is responsible for the split of the basal pole [14,15]. The critical resolved shear stresses (CRSSs) of non-basal slips (such as the pyramidal <**c** + **a**> slip) decrease substantially with the increase of the temperature [16]. The CRSS of the pyramidal slip at room temperature is about 100 times larger than that of the basal slip [17]. This value decreases as the temperature rises, which means that the non-basal slip is much easier activated at elevated temperatures [18,19]. As such, it can be deduced that the AZ31 alloy sheet processed by Route C at 200 °C exhibits a tendency of splitting probably on account of the activation of the non-basal slips here.

**Figure 6:**
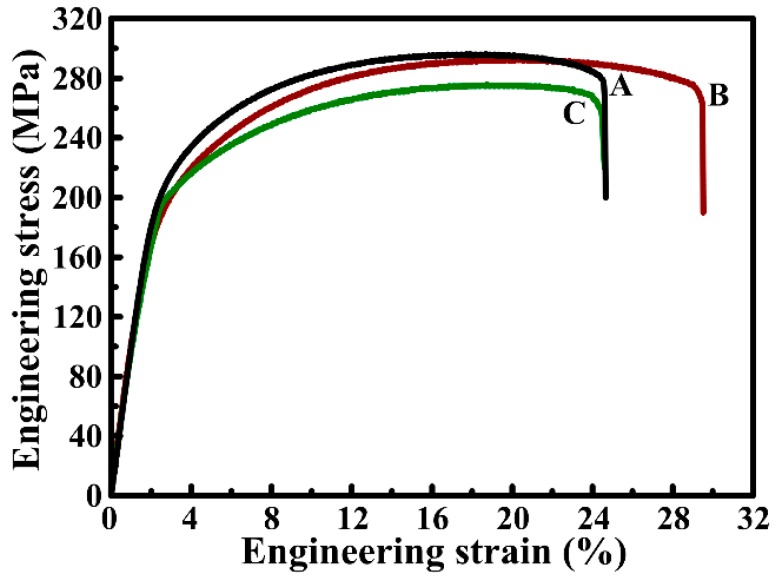
Tensile engineering stress–strain curves AZ31 Mg alloy sheets processed by (**A**) Route A; (**B**) Route B; and (**C**) Route C, respectively.

Tensile engineering stress–strain curves of the as-annealed AZ31 alloy sheets processed by the three methods are plotted in Figure 6. Average tensile properties are presented in Table 1, which includes the yield strength (σ_0.2_), ultimate tensile strength (σ_b_), elongation-to-failure (δ_f_), and plastic elongation (δ_P_). The σ_0.2_ and σ_b_ for Route A and B are nearly identical with actual errors. Note that the δ_f_ and δ_P_ obviously increase from 26.7% and 23.3% to 30.9% and 28.9%, respectively. However, the mechanical properties of the AZ31 alloy processed by Route C decreases compared with the ones of Route B. Therefore, the AZ31 alloy sheet processed by Route B presents excellent integrated mechanical properties among the three rolling routes. Table 2 shows tensile properties of some rolling AZ31 alloy in literatures [14,20,21,22,23,24,25,26,27,28]. It can be found that the AZ31 alloy sheet processed by Route B also shows excellent integrated tensile properties compared with the reported rolling AZ31 alloys.

The strength of the AZ31 alloy processed by both Route A and B are nearly identical, although the texture of Route A alloy has been weakened. Therefore, it can be deduced that the strength of the Route B alloy benefits from the finer grain size compared with the Route A alloy. It should also be noted that the δ_P_ is observably improved by Route B. In general, conventional rolled Mg sheets present a typical basal texture where most basal poles are parallel to the sheet plane [20,21,22]. In this case (RD tension), strain localization and premature shear failure occur on account of the suppression of the basal slip with the Schmid factor (SF) of zero [6,23]. Therefore, the excellent δ_P_ can be attributed to the weakened basal texture besides the fine grains in the AZ31 alloy produced by Route B. Conversely, the mechanical properties of the AZ31 alloy rolled by Route C decrease compared with the ones rolled by Route B, which can be caused by coarse grains in the alloy (Figure 3c). Therefore, excellent mechanical properties can be obtained by Route B due to the fine grains with weakened basal texture.

## 4. Conclusions

In the present study, the effects of three rolling routes on the microstructure and tensile properties of twin-roll casting AZ31 Mg alloy sheets were investigated. The grain size of the as-annealed AZ31 alloy processed by Route A (unidirectional-rolling), B (head-to-tail rolling), and C (clock-rolling) is 4.4, 4.0, and 7.3 μm, respectively. The basal texture intensity is 15.1, 13.4, and 14.4 for the Route A, B, and C, respectively. Route B is the most effective at refining the microstructure and weakening the basal texture among the three rolling routes. The AZ31 alloy sheet processed by Route B presents excellent integrated tensile properties. The corresponding σ_0.2_, σ_b_, δ_f_, and δ_P_ are 196 MPa, 301 MPa, 30.9%, and 28.9%, respectively. The tensile strength can benefit from the fine grains of the AZ31 alloy processed by the head-to-tail rolling route, while the excellent plastic elongation is achieved owing to the weakened basal texture besides the fine grains.

## Figures and Tables

**Table 1:** Tensile properties of the as-annealed AZ31 alloy sheets processed by the three rolling methods at room temperature.

Route	σ_0.2_/MPa	σ_b_/MPa	δ_f_/%	δ_P_/%
A	$199_{-8}^{+5}$	$298_{-2}^{+2}$	$26.7_{-2.1}^{+2.7}$	$23.3_{-1.3}^{+2.3}$
B	$196_{-1}^{+1}$	$301_{-9}^{+13}$	$30.9_{-1.3}^{+0.7}$	$28.9_{-1.1}^{+0.7}$
C	$192_{-7}^{+8}$	$280_{-5}^{+4}$	$25.8_{-1.2}^{+1.2}$	$24.0_{-1.4}^{+0.8}$

**Table 2:** Tensile properties of rolling AZ31 alloy in the literature.

Alloy	Grain Size (μm)	σ_0.2_/MPa	σ_b_/MPa	δ_f_/%	δ_P_/%
AZ31 [14]	10.2	161	272	19.7	~
AZ31 [24]	10	~	273	~	8
AZ31 [25]	~	250	295	~	16.2
AZ31B [26]	7.2	167	263	25.8	
AZ31 [27]	~	254	320	~	13.0
AZ31 [28]	~	180	270	19%	~
AZ31 [29]	7.4	158	280	20.3	~
AZ31 [30]	13	147	306	27.3	~
AZ31 [31]	3~20	175	277	~	21
AZ31 [32]	2.8	290	~310	~	23
